# The ultimate power play in research - partnering with patients, partnering with power

**DOI:** 10.1186/s40900-025-00745-9

**Published:** 2025-06-17

**Authors:** Dawn P. Richards, Janelle Bowden, Patrick Gee, Alex Haagaard, Anita Kothari, Annette McKinnon, Codie A. Primeau, Andrea C. Tricco, Ellen Wang, Karen L. Woolley, Linda C. Li

**Affiliations:** 1 Canadian Institutes of Health Research Institute of Musculoskeletal Health and Arthritis, University of British Columbia, Vancouver, BC Canada; 2 Five02 Labs Inc., Toronto, ON Canada; 3 Patient Author, Toronto, ON Canada; 4 AccessCR Pty Ltd, Sydney, NSW Australia; 5 University of Queensland, Brisbane, QLD Australia; 6 iAdvocate, North Chesterfield, VA USA; 7 Patient Author, Kingston, ON Canada; 8 DigiMSK Research Group, Center for Aging SMART, Vancouver Coastal Health Research Institute, Vancouver, BC Canada; 9 School of Health Studies, Western University, London, ON Canada; 10 Canadian Arthritis Patient Alliance, Toronto, ON Canada; 11 Patient Advisors Network, Toronto, ON Canada; 12 Arthritis Research Canada, Vancouver, BC Canada; 13 Department of Physical Therapy, University of British Columbia, Vancouver, BC Canada; 14 Li Ka Shing Knowledge Institute, St. Michael’s Hospital, Unity Health Toronto, Toronto, ON Canada; 15 Epidemiology Division, Institute of Health Policy, Management and Evaluation, Dalla Lana School of Public Health, University of Toronto, Toronto, ON Canada; 16 University of British Columbia, Vancouver, BC Canada; 17 Caregiver Author, Australia; 18 University of the Sunshine Coast, Sippy Downs, QLD Australia

**Keywords:** Power dynamics, Power imbalance, Power, Patient and public involvement, Patient engagement, Consumer involvement, Patient partnership, Research, Co-production, Diversity, Equity, And inclusion

## Abstract

**Background:**

Patient and public involvement (PPI), also called patient engagement, patient partnership, or consumer involvement, holds potential to change approaches and outcomes in research and healthcare. All research teams have complex power dynamics, including those with patient/public members. We present our perceptions and understandings of power arising from our own experiences on health research teams. We suggest ways for members of health research teams to move forward in efforts to minimize power discrepancies.

**Main body:**

As an international group of patients, caregivers, and research allies, we have experienced power dynamics within PPI collaborations and believe they must be challenged to achieve more equitable partnerships. We explore four themes relating to power in no order of importance: (1) The unstable and changing nature of power in PPI. Patient/public partners’ abilities and capacities to engage equally depend on the working environment and on their economic, cultural, social and symbolic (including health) capitals; (2) Power between and amongst patients/public partners. Layers of power exist between and amongst patient/public partners and their networks, which may lead to a lack of diversity in partners and/or bullying and requires recognizing that not all patient/public partners bring the same experiences, skills or perspectives to research teams; (3) Power and tokenism. Tokenism occurs when patient/public perspectives in PPI are mostly ignored, results when power and resources are disproportionately concentrated, and can be perpetuated by funding and funding agency infrastructures; and, (4) PPI as a commodity or product. PPI may be seen or used as a means to extract experiences or validate one’s work without truly involving patients/public contributors in the research design and process. PPI aligns with a broader trend of academic research methodologies grounded in standpoint epistemology (that is, how a person’s social identity influences what they know). We include practical recommendations for researchers and for patient/public partners to share power more equitably on research teams.

**Conclusion:**

In our experiences on health research teams, patient/public partners are often the most vulnerable and most disadvantaged members of the team who experience the largest power inequities. We hope our identified themes about power, the context related to power, and our reflections and recommendations on them inspire those holding power on research teams to share that power.

## Abstract (plain language summary)

Patient and public involvement (PPI) may also be called patient engagement, patient partnership, or consumer involvement, and can change the ways research is done and how healthcare is delivered. All research teams have dynamics related to who holds power, including teams with patient/public members. We are an international team that includes patients, caregivers, and research allies. We have experienced power dynamics within our PPI collaborations and believe these power dynamics must be challenged so better partnerships can be built. We look at four themes relating to power in no order of importance. First, power is unstable and changes in PPI. For example, patient/public partners’ abilities and capacities to contribute are greatly affected by where the work is done and their economic, cultural, social backgrounds. Second, there are power dynamics between and amongst the patients/public partners. These may lead to a lack of diversity in partners and/or bullying and requires recognizing that not all patient/public partners bring the same experiences, skills or perspectives to teams. Third, tokenism is when patient/public perspectives in PPI are mostly ignored, and is often a result of how research is funded, including who holds the funding. Lastly, PPI is sometimes seen as a commodity or product used to benefit the research team, but not necessarily the patients/public contributors. Context is also an important factor in the discussion about power. Based on our own experiences, we suggest ways for members of health research teams to more equitably share power.

## Background

Patient and public involvement (PPI), also known as patient engagement, patient partnership, or consumer involvement, holds vast potential in research and healthcare for innovative approaches and improved outcomes [1]. True partnership shifts the dynamic of patient/public members from simply having a seat at the table to having power in decision-making processes (i.e., shared ownership of the table) and is imperative as the benefits of PPI become clearer [2–6]. Having a seat at the table is a metaphor for an imbalance of power. Individuals with a seat at the table are there at the request of the person who or organization that owns the tables [7, 8].

While power can be “possession of control, authority, or influence over others” [9] we also examine the exercise of that power. In this paper “…what characterizes the power we are analyzing is that it brings into play relationships between individuals (or between groups)…The term “power” designates relationships between partners.” [10] Power relations or dynamics are the ways people access and use power in their interactions with each other [11]. Power dynamics in research and healthcare teams are complex and contextual. They impact relationships and interactions of all members of the team in different ways, including those between and amongst: patient/public partners and health researchers, researchers and their researcher peers, and patient/public partners and their own peers. These power dynamics may be explicit (e.g., using titles) or implicit (e.g., our biases). Currently PPI in research is often idealized in funding applications, reports, and publications, while we know that unequal or inequitable access to funding, time, and resources is a source of power disparities [12, 13]. These imbalances are made possible by the existing structures and culture of research, often rooted in exclusionary and structurally unjust practices [14–16]. This may lead to interpersonal conflict, stress, burnout, and traumatization (or re-traumatization) [8]. The aim of this paper is to present our perceptions and understandings of power arising from our experiences on health research teams. We also suggest ways for members of health research teams to move forward in efforts to achieve a more equitable distribution of power.

The idea for this team, exploring power in PPI, and this Commentary paper, evolved from discussions started at the PxP Conference in 2023 between DPR and LCL [17]. LCL undertook an abbreviated literature review on this topic, and created and shared a document with a summary of findings and some themes that emerged with DPR. LCL and DPR had a discussion centred around this document, added thoughts to it, and co-developed a plan to invite others to the team. When others were invited and agreed to be part of the team, they also received a copy of this summary document. During a virtual discussion, several team members reflected on the implications of power in PPI informed by their own experiences and insights, and grounded in the literature, having been provided the same document that LCL and DPR originally discussed. Other team members were invited based on their diverse: experiences in PPI, perspectives related to research (patient or caregiver partner, trainee, researcher), sexes, genders, sexual orientations, ethnicities, and geography. That virtual meeting and subsequent virtual interactions (email and Google Docs) contributed to this work, including an opinion piece submitted to The BMJ for its July 2024 Patient Led issue. Team members were encouraged to contribute to conversations and writing in ways that worked for them, with everyone’s perspectives and suggestions being incorporated into this paper. Reflections and comments from one individual on the team spurred other reflections and comments in the form of an email trail or tracked edits or comments on a single document that was circulated. Patient and caregiver partners were offered honoraria offered through LCL’s research funds (noting that DPR identifies as a patient partner who lives with a chronic condition and was supported through their role with one of the Canadian Institutes of Health Research institutes). They were also supported by other means to take the lead on developing and fleshing out ideas. We attempted to share power and even invert its hierarchy as much as possible in our project, for example, LCL explicitly supported DPR to lead the writing and to co-lead calls and other communications with the team.

## Main text

As an international group of patients, caregivers, and research allies, we have experienced power dynamics within PPI research collaborations. We recognize that these teams are working within complex sociotechnical ecosystems that include funding bodies, and organizational and academic norms. These ecosystems have the ability to impact on a team’s internal power dynamics – in both positive and negative ways [18, 19]. For instance, the prominence of PPI in research emerged in part from sustained efforts by funding agencies promoting partnerships between researchers and patients [20–22]. This reflects a longstanding recognition of the real-world challenges in health research, such as limitations in study designs, difficulty in translating findings into practice, and misalignment with patient priorities [23, 24]. We also realize that dynamics resulting from healthcare may also spill on to research teams. Healthcare is riddled with power between healthcare providers and patients [25], which may impact the perspectives and behaviours these individuals then bring to a research environment [26]. In particular, patients may have experienced trauma, harm, marginalization, and more, and may have been made even more vulnerable.

**Fig. 1:**
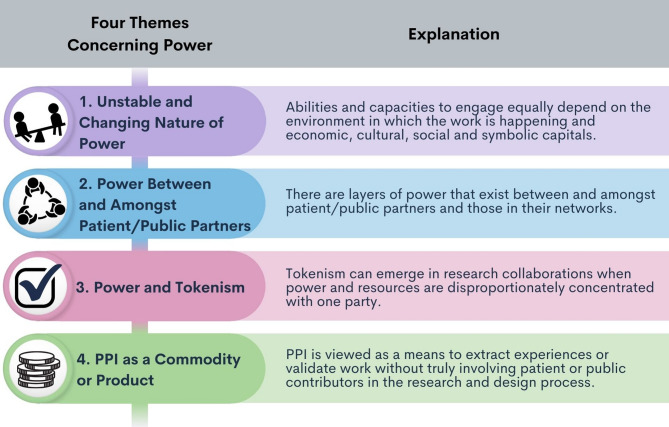
Four themes concerning power in PPI. The figure shares and describes four themes our team has identified relating to power in PPI. These themes are not presented in any order of importance

Given our own lived experiences, we focus on challenging the power dynamics of PPI on health research teams to enable more equitable partnerships. This article delves into four themes concerning power in no order of importance: (1) the unstable and changing nature of power in PPI, (2) power between and amongst patient/public partners, (3) power and tokenism, and (4) PPI as a commodity (Fig. 1). Further, we offer practical recommendations based on our collective learnings from partnerships where power was and was not shared.

### Themes concerning power

#### 1. The unstable and changing nature of power

Patient/public partners’ abilities and capacities to engage equally depend on the environment in which the work is happening (e.g., academic, healthcare, or other setting), and their economic, cultural, social and symbolic capitals (e.g., their illness experience or their knowledge about a condition [27]). However, individuals’ power is unstable [27]. For instance, hermeneutical injustices are disempowering [28]. Hermeneutical injustice is where individuals lack the language to articulate their own experiences in a way that is understood by those in power [29]. Certain health conditions are viewed and judged with varying degrees of legitimacy and stigma by those in power (e.g., being diagnosed with cancer vs. fibromyalgia or myalgic encephalomyelitis) [30]. Moreover, individuals may be questioned for their legitimacy to contribute to research through their lived experiences after they acquire formal academic research training [27]. Recognizing these potential threats to equitable power distribution are critical for patient/public members, research allies, and other parties to engage in genuine partnerships.

As patients move through living with a health condition, their power and abilities as a patient partner also change, and some change more than others. Shoemaker et al., describe the process to becoming a patient partner in research as one that occurs through adversarial growth [31]. The four stages include lived experience, personal story, collective perspective, and patient research partner. These stages are characterized by varying abilities to contribute as a patient partner, and which then also mean different types of abilities and subsequent power. For example, the first 2 stages are where patients are best able to represent their own experiences and stories, while when and if they move to the latter stages, they know more about their peers’ and communities’ perspectives and about research as well [31]. Not all patient partners move beyond the first or even subsequent stages, and this movement (or lack of) impacts their abilities to contribute, influence, and add what they know about others’ situations [31]. For example, people who are in later stages may also be able to influence in other ways, for example helping mentor trainees relating to PPI or even be called on to teach the research team more about PPI [32].

#### 2. Power between and amongst patient/public partners

While we have primarily focussed on how researchers need to be more informed about and aware of power and how they can share it, we also recognize there are layers of power that exist between and amongst patient/public partners and those in their networks. Some patient/public partners are often invited to be part of PPI collaborations or are well-known as partners. This may result in some patient/public partners being ‘invited to the table’ more often than others who have equally as important and distinct perspectives, but who are not as well-known or do not possess such a large network. This may result in these individuals helping bring others ‘to the table,’ while patient/public partners ‘at the table’ may still lack diversity (for example, patient partners who co-author publications with academic researchers are primarily female, middle-aged, from high-income countries, publishing in English [33]). We support meaningful long-term partnerships with patient/public partners, and caution that these partnerships should not limit efforts to involve a diversity of lived experiences and skills. Lastly, those who get invited to the table as representatives of marginalized perspectives (for example, those that have been historically exploited, harmed, or discriminated against in healthcare and medicine) often hold a lot of power in relation to the marginalized communities they are invited to represent. Diverse engagement is essential to rebuild trust that has been lost with these communities and individuals from these communities need to be at the table to make sure the work reflects the needs of their communities.

We note that bullying can and does occur in the patient/public partner space – these individuals are human after all [34]. While often not discussed openly, patient/public partners may bully their peers or they may bully other members of the research team. In some instances, patient/public partners may not realize or understand that others have experiences different from their own or they generalize their own experiences to others, representative of being in one of the first two stages (“lived experience” or “personal story”) of Shoemaker et al.’s description of being a patient partner [31]. When these individuals do not feel their perspective is heard or reflected in a project, they can view the experience through a lens of oppression or being slighted. And while the resulting response or reaction may stem from frustration for not having their preferences prioritized, it can also be interpreted as them simply acting out for not getting their own way. Examples of such unprofessional behaviours range from uncomfortably confronting researchers and other team members to silencing other patient/public partners. These individuals’ behaviours can affect all members of the team in a variety of ways and may have lasting consequences. For example, research team members, including patient partners, may be dissuaded from engaging in PPI. Bullying may also manifest through forms of discrimination, which may discourage individuals from underrepresented communities from engaging as partners.

This area points to the need for research teams to consider the ‘fit’ in their working together. Just like any other team, it is important to have the right skills, expertise and perspectives on the team to improve the team experience and outcomes for the research and the team. It is critical to recognize that all patient/public partners do not bring the same skills or perspectives to research teams [31]. Understanding patient/public partners’ skills as well as where they are in their own partner ‘journey’ in terms of representing their own perspectives or also being knowledgeable about others is valuable [31]. In fact, some individuals with lived experience may serve to help research teams navigate and understand the nature of power dynamics among patient/public partners or communities [34]. Sometimes an intermediate organization that works with the community can assist with understanding the patient partners’ perspectives (e.g., explaining historical injustices). If asking the patient/public partners on the team, this type of sensitizing role for patient/public partners may help them get the most out of their own skills while also supporting research teams to develop and manage relationships that are true to the ideal of PPI in research.

#### 3. Power and tokenism

In its simplest form, tokenism occurs when patient/public perspectives in a PPI collaboration are mostly ignored [35]. Tokenism can emerge in research collaborations when power and resources (e.g., funding, leadership, etc.) are disproportionately concentrated with one party, usually institutionally-affiliated researchers. For example, patients/public partners may not be expected or welcomed to contribute, may not receive needed support to meaningfully contribute (e.g., compensation or other resources) and may be offered only limited opportunities related to making decisions around their own roles, and project goals, scope and methodology. Tokenism can be thought of as a superficial approach to engagement.

This imbalance can be reinforced or perpetuated by funding and funding agency infrastructures. There is currently a lack of standardized or required training for researchers related to PPI, with many learning to do PPI ‘on the fly’ [36, 37]. Additionally, some funding opportunities that require PPI may inadvertently incentivize tokenistic approaches to PPI. There are few funding opportunities designed specifically to support PPI team relationship building, for example, in advance of fully developing a project idea or submitting a larger grant application [38]. As a result, many patient/public partners are not properly supported and may even incur financial and emotional costs for efforts they expend contributing to a project’s development before it is stably-funded. Further, some patient/public team members may not even be invited to join a project team until it is funded, by which time the project has already been designed without their insights and input [39].

There are limited opportunities for patient/public partners or patient organizations to apply for or hold funding [40]. Moreover, institutional funding opportunities that are explicitly oriented to health equity and community engagement often have narrow or confusing criteria that limit the eligibility of patient partners with diverse, uncertain or complex diagnoses, or those with non-academic backgrounds or without organizational affiliations, and exclude these individuals from co-leadership roles on the team. One possible consequence of this is that individual patient and public partners may not be supported for their time, skills, and expertise (with compensation or additional supports). While changing rules related to funding may (or may not) be difficult for many reasons, understanding what patient and public partners need as supports to be part of a team is relatively easy to determine.

Tokenistic PPI can lead to harms for patient/public partners. These partners can be exposed to intersecting forms of systemic oppression (racism, homophobia or transphobia, ableism, and classism, among others) and disparagement of their identities and lived experiences, both from other members of the research team and from the broader institutions in which they work [41]. The intersecting and evolving identities of individuals on a PPI collaboration also shape the power dynamics [42].

Ongoing and open conversations are required to understand how patients/public partners wish to contribute and their needs in doing so, and to promote a safe and inclusive team environment with supports needed for meaningful engagement [43]. As with any team diverse in skills, interests, perspectives and experiences, building a relationship on a PPI team takes time, energy, and conversation so that people can better understand each other and the basis of their perspectives [43].

#### 4. PPI as a commodity or product

PPI is viewed by some researchers as a means to extract experiences or validate their work without truly involving patients/public contributors in the research and design process [44]. Unfortunately, this can lead to a superficial, transactional, and exploitative relationship where patient/public partners are present to simply tell their stories (which in themselves are important), agree to the rest of the team’s decisions, or provide ‘credibility to a grant application or project’. While patient and public members bring experiences that are different from other members of the team, they also bring their own skills, expertise, and ideas. They bring value to the entire project, not just their ‘slice of experience’ as a patient or caregiver [45]. Rather than setting up relationships to simply extract ‘parts’ from people, relationships should be built from a place where everyone is equal [15, 46]. A team that values all members’ unique skills, expertise, experiences, and abilities to contribute beyond their personal experiences fosters a more collaborative and inclusive dynamic [47]. Team members should regularly self-reflect on their positionality relative to others around the table in terms of how that may be influencing power dynamics, the nature of discussions, and decision-making on the team [48].

PPI aligns with a broader trend of academic research methodologies, such as co-design, grounded in standpoint epistemology (i.e., how someone’s social identity affects what they know and how they understand the world) [49]. These methodologies (cl)aim to make research more equitable by engaging directly with marginalized and underrepresented communities and perspectives. However, decolonial critiques have pointed out how in practice, they are often extractive and exploitative [50–53]. We have observed that some organizations, institutions and researchers replicate this pattern of exploitation.

### Recommendations for more equitably sharing power

Based on our experiences, we have developed recommendations for more equitably sharing power. We present these as Table 1 and indicate who the recommendation is for, that is researchers (which also includes other research team members, excluding patient/public partners) or patient/public partners, when the recommendation should be done in terms of timing related to a research project, and which power theme(s) the recommendation is intended to impact (represented in the order they are numbered above).

**Table 1:** Recommendations to more equitably share power on research teams that include patient/public partners

Recommendation	Who this is for	When this should be done	Which power theme(s) this impacts
1. Consider the types of skills, perspectives, and experiences a patient/public partner or partners may bring to best support the project and complement the team.	Researchers	Before you bring patient/public partners on to the team	3, 4
2. Ask yourself if you have the appropriate experiences and perspectives to be part of the project team.	Patient/public partners	Before you join a team	3, 4
3. Reflect on your own positionality and current team power dynamics or working behaviours and consider how patient/public partners may perceive or experience them (48).	Researchers	Before you bring patient/public partners on to the team	4
4. Take cultural safety, cultural competency, and trauma-informed training to learn how to help create a safe space for trust building to occur.	Researchers	Before you bring patient/public partners on to the team	4
5. Budget sufficiently for outreach and supports, including compensation for patient/public partner time and efforts and accessible knowledge mobilization outputs and products. Budget for caregiver supports as well.	Researchers	Before you bring patient/public partners on to the team	3
6. Ask patient/public partners how they wish to contribute, in what capacity, and what supports they need (e.g., with technology, compensation, etc.). This may be challenging if there is no existing relationship or if partners do not know the possibilities. Providing a list of examples of suggestions may be helpful. Plan for flexibility and for the opportunity to readjust expectations if needed over time.	Researchers	Before you bring patient/public partners on to the team, and throughout a project	3, 4
7. Rather than simply extracting ‘parts’ from people, invest in building relationships where everyone is treated equally and regularly reflect on your own role and perspective (15, 46). Ideally building these relationships would happen well before research activities start.	Researchers	Before you bring patient/public partners on to the team, and throughout a project	4
8. Include and support patient/public partners from the start when there is time and room to adjust research questions, scope/framing, methodology, and terminology.	Researchers	At the start of a project	3, 4
9. Incorporate a realistic project timeline, accounting for life events (e.g., illness, work or otherwise) that might delay research activities.	Researchers	At the start of a project	3, 4
10. Proactively and sensitively check for the potential misuse of power on your team or within your patient/public partner community. For example, co-creating a PPI charter at the start of working together that covers the principles the team will follow or using an evaluation tool may be helpful (42, 43).	Researchers	At the start of and throughout a project	2, 3, 4
11. Demonstrate to patient/public partners how their contributions impact the project.	Researchers	Throughout a project	3, 4
12, Be aware of your own skills in managing conflict, communicating, advocacy, and other areas. Identify and seek out training and support (which may include peer support) to help you engage effectively.	Patient/public partners	Throughout a project	1
13. Regularly evaluate and reflect on your own representativeness of your community. This does not mean you need to or do represent everyone in your community, but it does mean being aware there are experiences and perspectives beyond your own (23).	Patient/public partners	Throughout a project	2
14. Be prepared for creative compromise, to get uncomfortable, and to have honest conversations to work through problems without easy solutions.	Researchers and patient/public partners	Throughout a project	3
15. When publishing, submit to journals that allow patient/public partners who meet authorship requirements and wish to be named, to be co-authors.	Researchers	Near the end of a project	3, 4
16. Advocate to research funders for better and earlier funding options for PPI, so as to better support true patient/public partnerships.	Researchers	Any time	3, 4
17. Consider working with research teams in another way if appropriate – such as helping them navigate the patient community, and helping them to find the appropriate patient/public partners for their work.	Patient/public partners	Any time	2
18. Work actively with key individuals across the research ecosystem, including at funding and healthcare organizations, and academic institutions, and other relevant entities to develop, support and sustain authentic partnerships between researchers and patient/public partners, and with institutional collaborators.	Researchers, patient/public partners, others in the research ecosystem	Any time	1, 2, 3, 4

Legend: Recommendations are numbered in keeping with plain language principles so they are easy to reference, rather than numbered to indicate priority order. Power themes are labeled by the numbers in which they appear in the main text: (1) Unstable and changing nature of power, (2) Power between and amongst patient/public partners, (3) Power and tokenism, (4) PPI as a product or commodity

## Conclusions

Power dynamics on any research team are complex, and including patient/public partners on the team adds to that complexity. In our experiences on health research teams, patient/public partners are often the most vulnerable members of the team who experience the largest power inequities. Here we identify four themes related to PPI and power on health research teams: power is unstable and changes in PPI, there is power between and amongst patient partners, power and tokenism are closely related, and PPI as a commodity. A research team’s own power dynamics may be influenced by the context in which it works, just as that team may also influence the external power dynamics in which it operates. We offer reflections and recommendations related to these themes to help recognize where and how power imbalances can exist, and what might be done, when, and by whom to better minimize these. Ultimately, we hope to inspire the sharing of power to achieve greater success and impact from your PPI activities.

## Data Availability

No datasets were generated or analysed during the current study.

## Abbreviations

PPI: Patient and public involvement
